# Enhanced performance and reduced emissions in compression-ignition engine fueled with biodiesel blends synthesized via CaO and MgO nano catalysts

**DOI:** 10.1186/s13065-025-01604-0

**Published:** 2025-08-13

**Authors:** Sabah Mohamed Farouk, Aghareed M. Tayeb, Shereen M. S. Abdel-Hamid, Randa M. Osman, Hassan M. M. Mustafa

**Affiliations:** 1 Egyptian Academy for Engineering and Advanced Technology (EA&EAT), Affiliated to the Ministry of Military Production, Chemical Engineering Department, Km. 3 Cairo Belbeis Desert Rd., Cairo Governorate, 3066 Egypt; 2 Faculty of Engineering, Minia University, Misr Aswan Agricultural Rd., EL MAHATTA, Menia Governorate, 2431384 Egypt; 3 Chemical Engineering and Pilot Plant Department, National Research Centre (NRC), 33 El Bohouth St, Dokki, Giza Governorate 12622 Egypt; 4 Mechanical Engineering Department, Engineering and Renewable Energy Research Institute, National Research Centre, Giza, Egypt

**Keywords:** Waste cooking oil, Biodiesel, Nano-catalysts (CaO and MgO), Engine performance, Exhaust emissions

## Abstract

The rapid depletion of fossil fuel reserves has intensified the pursuit of sustainable alternatives for compression-ignition engines. Biodiesel, produced from renewable feedstocks such as waste cooking oil (WCO), offers an environmentally benign substitute that can be utilized either neat or in blends with conventional diesel. This study examines the comparative efficacy of nano-calcium oxide (CaO) and nano-magnesium oxide (MgO) catalysts in synthesizing biodiesel from WCO, emphasizing their physicochemical characteristics and subsequent effects on diesel engine performance and emissions. Distinct from prior investigations that focused on single catalysts or lacked engine-level validation, this work integrates detailed catalyst characterization (SEM, BET, XRD) with comprehensive combustion testing of various biodiesel blends. The synthesized biodiesel was blended with petroleum diesel at volumetric ratios of B10, B20, and B30 and evaluated in a single-cylinder diesel engine. Characterization results demonstrated superior catalytic activity of nano-CaO (average particle size: 67.1 nm; specific surface area: 80.7 m²/g) compared to nano-MgO (32.5 nm; 60.2 m²/g). Engine performance testing revealed that the NC(CaO)B10 blend reduced brake-specific fuel consumption by 8.3% and improved thermal efficiency at 75% engine load relative to baseline diesel. Furthermore, NC(MgO)B30 lowered CO₂ emissions by 4.2%, whereas NC(CaO)B30 achieved a 0.7% reduction in CO emissions and an approximate 3% increase in excess oxygen availability. These findings underscore the potential of CaO-based Nano catalysts, particularly at lower blend ratios, to enable cleaner and more efficient diesel engine operation. This work advances the case for nanotechnology-enhanced biodiesel as a viable component of sustainable fuel systems and highlights opportunities for optimization through higher blend ratios and synergistic catalyst combinations.

## Clinical trial number

Not applicable.

## Introduction

The accelerating depletion of petroleum resources, escalating fuel consumption, and increasingly stringent environmental regulations present significant challenges to the global energy sector. The development and commercialization of bioenergy offer a promising pathway to mitigate both petroleum dependency and associated environmental impacts [1]. Among the various biofuels, biodiesel has emerged as a renewable, sustainable, and technically viable alternative for compression-ignition (CI) engine applications [2–4].

Diesel engines, widely employed in transportation and industrial sectors, have been extensively studied for performance and emissions when fueled with biodiesel and its blends. Biodiesel is particularly attractive due to its lower emissions of hydrocarbons, carbon monoxide (CO), and particulate matter compared to conventional diesel, making it one of the most environmentally favorable alternative fuels.

Transesterification remains the most efficient and widely adopted method for biodiesel production, involving the reaction of triglycerides from vegetable oils with an alcohol (typically methanol) to yield fatty acid methyl esters (FAME) and glycerol as a byproduct. Glycerol itself has notable value in the pharmaceutical and cosmetic industries [4]. Several key parameters influence the transesterification process, including reaction temperature, alcohol-to-oil molar ratio, moisture and free fatty acid content, mixing intensity, alcohol type, reaction duration, and catalyst concentration [5–7]. Catalysts used for transesterification are generally classified as homogeneous, heterogeneous, or biocatalysts [8, 9].

Nanocatalysts have gained considerable attention due to their superior catalytic efficiency, reusability, and ease of separation from the final product. In previous studies, techniques such as Scanning Electron Microscopy (SEM), X-Ray Diffraction (XRD), and Brunauer–Emmett–Teller (BET) surface area analysis have been extensively employed to characterize nanocatalysts by examining their morphology, crystallinity, and surface area, respectively [10–12]. These techniques provide vital insights into active surface properties and nanoscale structure, directly influencing catalytic activity. In the present study, we applied these tools to validate the structural and surface characteristics of CaO and MgO nanoparticles, linking their physicochemical features to catalytic performance [10–12]. In this study, these techniques were applied to evaluate the structural and surface properties of CaO and MgO nanoparticles and correlate their physicochemical characteristics with catalytic efficiency.

Among heterogeneous catalysts, calcium oxide (CaO) and magnesium oxide (MgO) nanoparticles are particularly promising owing to their strong basicity, low toxicity, thermal stability, and reusability [13]. Compared to other systems, such as ZnO or KOH-based catalysts, CaO and MgO offer additional advantages, including non-corrosiveness, cost-effectiveness, and environmental compatibility [10–12]. Although previous studies have demonstrated the potential of each catalyst individually, few have directly compared their nanoscale catalytic effects on both biodiesel synthesis efficiency and diesel engine emissions under identical conditions. This study addresses this gap by synthesizing and evaluating both Nano catalysts side-by-side, providing a comprehensive assessment of their performance.

Combustion of biodiesel, compared to petroleum diesel, typically results in reduced emissions of unburned hydrocarbons, CO, and sulfur dioxide due to its oxygenated nature and lower sulfur content. However, biodiesel blends tend to exhibit increased exhaust gas temperatures, slightly higher brake-specific fuel consumption, and marginal reductions in power, torque, and thermal efficiency. Furthermore, biodiesel combustion generally produces higher carbon dioxide (CO₂) emissions, but lower CO emissions compared to diesel [14]. Nevertheless, biodiesel’s carbon-neutral lifecycle contributes to reduced greenhouse gas emissions and improved air quality.

In this work, biodiesel was synthesized from waste cooking oil (WCO) via transesterification catalyzed by nano-CaO and nano-MgO. The resulting biodiesel was blended with petroleum diesel at volumetric ratios of B10, B20, and B30 and tested in a single-cylinder diesel engine. Key engine performance parameters—including exhaust gas temperature, thermal efficiency, volumetric efficiency, and air–fuel ratio—were evaluated alongside emissions of CO, CO₂, and O₂. The use of WCO as a feedstock enhances the sustainability of biodiesel production while addressing environmental concerns related to its improper disposal.

Most previous investigations have focused on either homogeneous catalysts or bulk metal oxides, often overlooking the impact of nanoscale properties on catalytic performance and failing to evaluate engine behavior under practical operating conditions. Few studies have critically compared nano-CaO and nano-MgO under a unified experimental framework using WCO as the feedstock. This study bridges this gap by correlating catalyst morphology and surface area with biodiesel yield, engine performance, and emission characteristics, offering a more holistic evaluation.

The main objectives of this research are: (i) to synthesize and characterize nano-CaO and nano-MgO catalysts using SEM, BET, and XRD; (ii) to evaluate their catalytic performance in transesterifying WCO into biodiesel; and (iii) to assess the engine performance and emissions of biodiesel blends (B10, B20, B30) produced with these catalysts. This integrated study aims to identify the most effective Nano catalyst and blending strategy to achieve cleaner and more efficient CI engine operation.

## Experimental section

### Materials

Waste cooking oil (WCO), consisting of a mixture of palm and sunflower oils, was collected from household sources.

All reagents were of analytical grade and used as received without further purification. Methanol (≥ 99.8%), magnesium sulfate (MgSO₄, ≥ 99%), calcium chloride (CaCl₂, ≥ 96%), and sodium hydroxide (NaOH, ≥ 98%) were supplied by Alpha Chemika Pharmaceuticals and Chemicals, Egypt.

### Method

#### Catalyst characterization

##### Scanning electron microscopy (SEM)

The morphology of the synthesized catalysts was examined by scanning electron microscopy (SEM), which provides high-resolution images of surface topography via electron beam scanning [15]. SEM micrographs were acquired using a QUANTA FEG250 microscope (FEI, USA) at the Egyptian National Research Centre (NRC).

##### Brunauer–Emmett–Teller (BET) surface area analysis

The specific surface area of the catalysts was determined using the Brunauer–Emmett–Teller (BET) method [16]. Nitrogen adsorption isotherms were measured at 77 K using a Quantachrome NovaWin 3200 surface area analyzer (USA). Prior to measurement, the samples were degassed at 120 °C under vacuum overnight to remove adsorbed moisture and contaminants.

##### X-ray powder diffraction (XRD) analysis

An X-ray diffraction (XRD) analysis was carried out to identify the crystalline phases present in the catalyst samples [17]. The measurements were performed using a PANalytical X’Pert PRO diffractometer equipped with CuKα radiation (λ = 1.540 Å), operated under standard conditions.

#### Biodiesel production processes

##### Nano catalyst synthesis

Calcium oxide (CaO) nanoparticles were synthesized via a controlled precipitation method. An aqueous solution of 0.5 M CaCl₂ was added dropwise to a 0.5 M NaOH solution under continuous stirring at 70 °C for 2 h. The resulting precipitate was filtered, thoroughly washed with deionized water, and dried at 110 °C for 12 h. The dried material was then calcined at 650 °C for 3 h in a muffle furnace [18].

Magnesium oxide (MgO) nanoparticles were synthesized using an analogous procedure, wherein 0.5 M MgSO₄ and NaOH solutions were reacted at room temperature under stirring for 2 h, followed by drying and calcination at 650 °C for 3 h [19]. All chemicals employed were of analytical grade and ≥ 99% purity (Alpha Chemika, Egypt).

The synthesized nanoparticles were subsequently characterized in terms of chemical composition, phase structure, morphology, and specific surface area.

##### Biodiesel production

The waste cooking oil (WCO) was first filtered to remove residual food particles, then heated to 110 °C for 30 min to eliminate moisture. Transesterification was carried out by mixing WCO with methanol and 1 wt% nano-catalyst (either CaO or MgO) under optimized conditions [20]. The specific reaction conditions were as follows:

- **CaO catalyst**: methanol-to-oil molar ratio of 6:1, reaction temperature of 70 °C, and duration of 85 min.
- **MgO catalyst**: methanol-to-oil molar ratio of 7:1, reaction temperature of 50 °C, and duration of 60 min.

All reactions were performed at atmospheric pressure with constant stirring at 500 rpm. Upon completion, the reaction mixture was transferred to a separating funnel and left undisturbed overnight to allow phase separation. The biodiesel layer was then washed twice with warm water (80 °C) to remove residual methanol and soap, followed by drying at 110 °C for 30 min. Catalyst residues were allowed to settle for 2–3 days [21].

For catalyst reusability assessment, the spent nano-catalysts were recovered by centrifugation, dried, and reused in subsequent transesterification cycles.

##### The biodiesel yield

The biodiesel yield was determined using Eq. (1) [22, 23]:

1 $$\begin{array}{l}\:Mass\:\,yield\:\% \:\\= \:\frac{{Actual\:\,weight\:\,of\,\:biodiesel}}{{Weight\:\,of\,\:oil\,\:used}} \times \,100\end{array}$$

The produced biodiesel was subsequently characterized for its key physical and chemical properties, including flash point, cloud point, kinematic viscosity, calorific value, and density, in accordance with ASTM standard testing methods.

##### Gas chromatography–mass spectrometry (GC–MS) and conversion efficiency

The chemical composition of the biodiesel was analyzed using a Shimadzu GC 17-A gas chromatograph coupled with a mass spectrometer. The system was equipped with a TG WAX-MS capillary column (30 m length, 0.25 mm internal diameter, 0.25 μm film thickness). The GC operated in split mode with a split ratio of 50:1, employing helium as the carrier gas at a constant flow rate of 1.0 mL/min. The injector temperature was maintained at 250 °C. The oven temperature program started at 50 °C (held for 2 min), then ramped to 250 °C at a rate of 10 °C/min. The MS detector operated in electron ionization (EI) mode at 70 eV, scanning in the m/z range of 50–550.

The conversion efficiency of waste cooking oil (WCO) to biodiesel was calculated using Eq. (2) [24]:

2 $$\begin{array}{l}\:Conversion\:\,\,efficiency\:\,\% \\= Ester\:\,content\,\:\% \:*\:Mass\:yield\:\%\end{array}$$

##### Preparation of biodiesel blends

Biodiesel–diesel blends were prepared volumetrically at specified mixing ratios. Two sets of blends were formulated based on the type of nano-catalyst used during biodiesel production:

- **CaO-based blends**:
  - NC (CaO) B10: 10% biodiesel + 90% diesel.
  - NC (CaO) B20: 20% biodiesel + 80% diesel.
  - NC (CaO) B30: 30% biodiesel + 70% diesel.
- **MgO-based blends**:
  - NC (MgO) B10: 10% biodiesel + 90% diesel.
  - NC (MgO) B20: 20% biodiesel + 80% diesel.
  - NC (MgO) B30: 30% biodiesel + 70% diesel.

All blended fuels were thoroughly mixed to ensure homogeneity prior to testing. These blends were subsequently evaluated for performance and emissions characteristics in a diesel engine and compared with conventional petroleum diesel.

#### Test of biodiesel in a diesel engine

A DEUTZ F1L511 single-cylinder, four-stroke, air-cooled, direct-injection diesel engine was employed to evaluate the performance and emissions characteristics of the biodiesel blends. The engine delivers a rated brake power of 5.775 kW at a constant speed of 1500 rpm. Engine load was incrementally varied from no-load to full load (0–75%) in four discrete steps corresponding to 0, 1, 2, 3, and 4 kW, using an electrical dynamometer.

Engine performance parameters and fuel consumption were measured using a calibrated precision burette–stopwatch arrangement. Exhaust gas temperatures were monitored continuously with K-type thermocouples, while emissions of O₂, CO, and CO₂ were quantified using an MRU DELTA 1600-V multi-gas analyzer. All instruments were calibrated prior to testing, with uncertainty margins of ± 1.5 °C for thermocouples, ± 2% for the pressure gauge, and ± 1% for O₂, CO, and CO₂ readings.

**Fig. 1:**
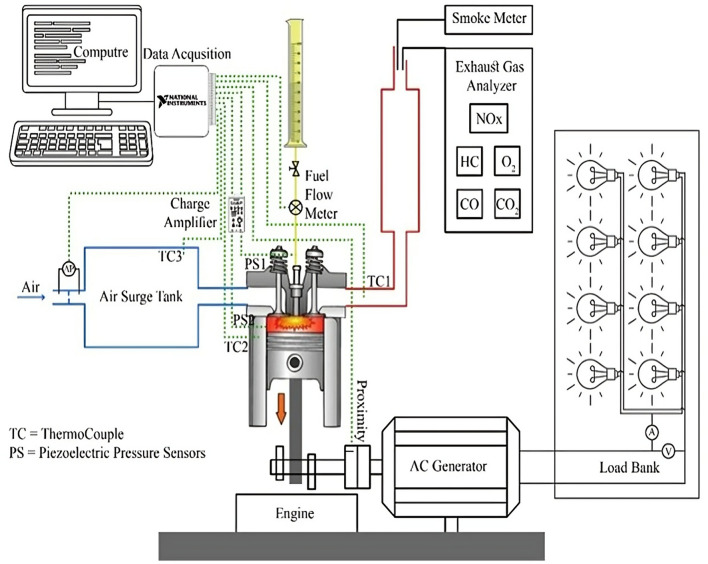
Diesel engine test rig schematic diagram

Each test condition was repeated three times, and the average value was reported to enhance reproducibility and reduce experimental error. The schematic of the experimental setup is presented in Fig. 1, and the key specifications of the diesel engine are summarized in Table 1.

**Table 1:**
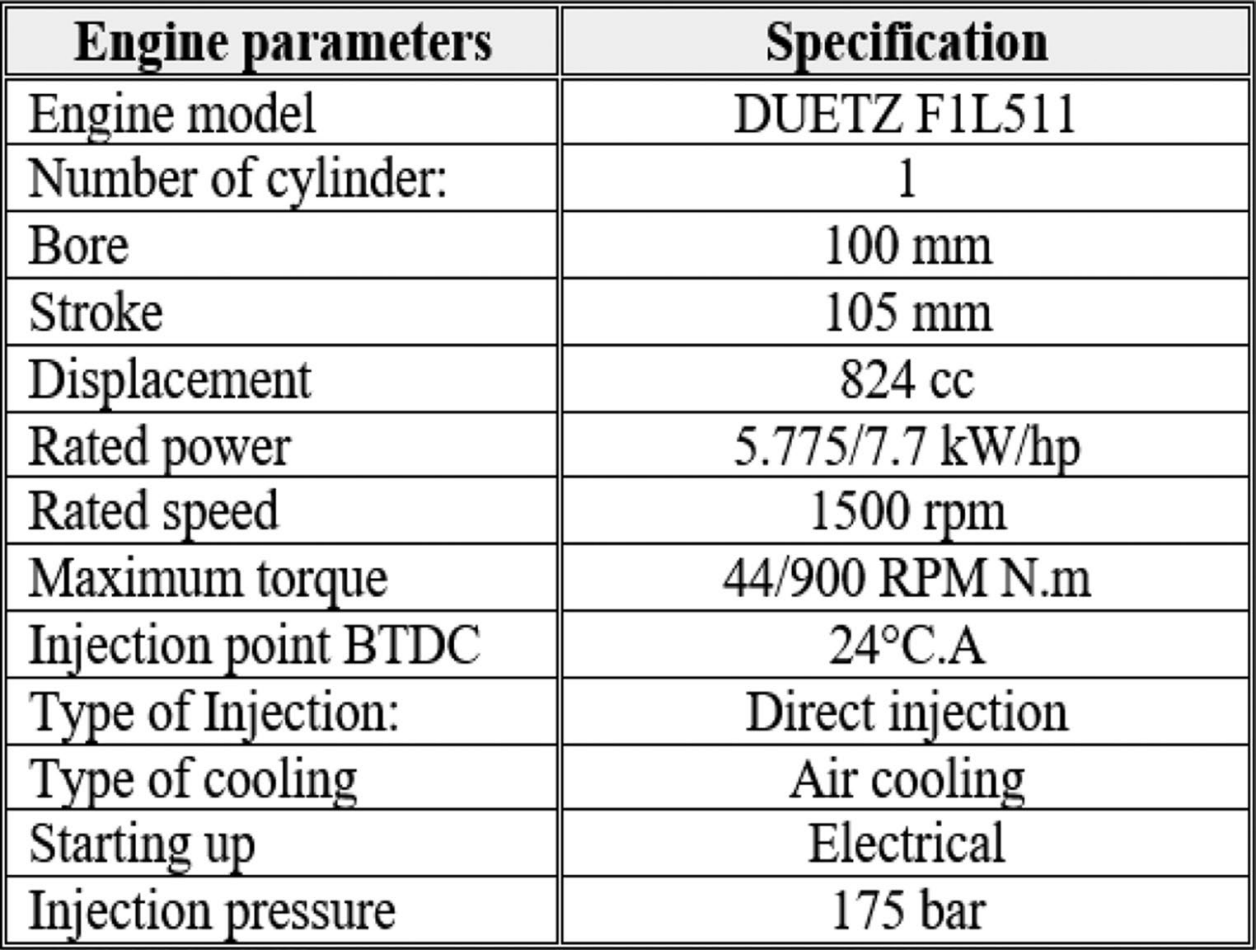
Diesel engine specifications

#### Performance parameters

##### Specific fuel consumption (SFC)

The specific fuel consumption (SFC) of the engine was determined using Eq. (3):

3 $$\begin{array}{l}\:Specidic\:\,fuel\:\,consumption\,\:\left({SFC} \right)\\= \:\frac{{Mass\,\:of\,\:fuel\,\:\left({\frac{{kg}}{h}} \right)}}{{Break\:\,power\:\,\left({kW} \right)}}\end{array}$$

where the mass of fuel consumed per hour was measured gravimetrically, and the brake power was determined from the dynamometer readings at each load condition.

##### Thermal efficiency

The thermal efficiency of the engine was calculated using Eq. (4):

4 $$\begin{array}{l}Thermal\:\,Efficiency\\= \frac{{Break\,\:power\: \times \:3600}}{{Calorific\:\,value\: \times \:Mass\:\,of\,\:fuel}}\end{array}$$

where brake power is expressed in kilowatts (kW), calorific value in kilojoules per kilogram (kJ/kg), and mass of fuel in kilograms per hour (kg/h). This ratio represents the proportion of the chemical energy of the fuel converted into useful mechanical work.

##### Air-fuel ratio (AFR)

The air–fuel ratio (AFR) was calculated to assess the combustion efficiency and its influence on emissions formation. The mass of intake air was measured and divided by the mass of fuel consumed under each operating condition to obtain the AFR.

##### Exhaust gas temperature

The exhaust gas temperature was continuously monitored using calibrated K-type thermocouples to evaluate the thermal response of the engine to different fuel blends and loading conditions. Measurements were recorded at each load step to capture variations attributable to fuel type and engine operation.

#### Exhaust emission parameters

Exhaust emissions were measured using an MRU DELTA 1600 V multi-gas analyzer to assess the environmental impact of biodiesel combustion relative to conventional diesel. The analyzer quantified the concentrations of carbon monoxide (CO), carbon dioxide (CO₂), and oxygen (O₂) in the exhaust stream under each operating condition.

##### Carbon monoxide (CO)

Measured as an indicator of incomplete combustion of hydrocarbons, typically elevated under fuel-rich conditions.

##### Carbon dioxide (CO₂)

Quantified to reflect the extent of complete combustion of hydrocarbons with oxygen.

##### Oxygen (O₂)

Monitored to evaluate the residual oxygen in the exhaust, noting that biodiesel inherently contains approximately 10% oxygen by mass, which contributes to improved combustion efficiency compared to petroleum diesel.

## Results and discussions

### Catalyst characterization

#### SEM-EDX analysis

**Fig. 2:**
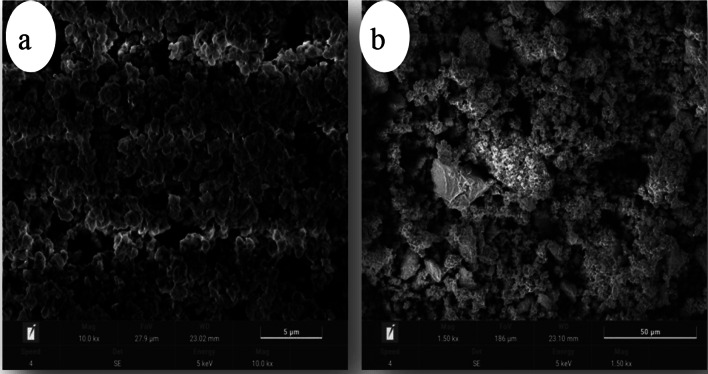
SEM micrographs of the synthesized (**a**) CaO nano-catalyst and (**b**) MgO nano-catalyst

Scanning Electron Microscopy (SEM) analysis was conducted at magnifications of 5 μm and 50 μm, as presented in Fig. 2. The SEM micrographs demonstrate that the synthesized CaO and MgO nano-catalysts exhibit irregularly shaped, porous morphologies, characterized by a high density of surface-active sites. Such structural features effectively increase the catalysts’ specific surface area, thereby facilitating enhanced transesterification performance by promoting more efficient contact between the reaction molecules and catalytic active centers.

#### BET surface area

The specific surface areas of the catalysts were determined using the Brunauer–Emmett–Teller (BET) method. As summarized in Table 2, the synthesized nano-CaO and nano-MgO exhibited substantially higher surface areas—80.69 m²/g and 60.2 m²/g, respectively—compared to their bulk counterparts, which recorded surface areas of 23 m²/g for CaO and 19 m²/g for MgO. This notable enhancement reflects nanoscale morphology, which increases the number of accessible active sites for catalytic activity. The enlarged surface area of the nano-catalysts facilitates more efficient reactions and interaction at the catalyst surface, thereby accelerating the transesterification reaction and improving biodiesel yield.

**Table 2:** Specific surface areas of bulk and nano-structured catalysts

Catalyst	Specific surface area (m2/g)	Reference
CaO	23	[25]
Nano-CaO	80.69	Present work
MgO	19	[26]
Nano-MgO	60.2	Present work

#### Powder XRD analysis

**Fig. 3:**
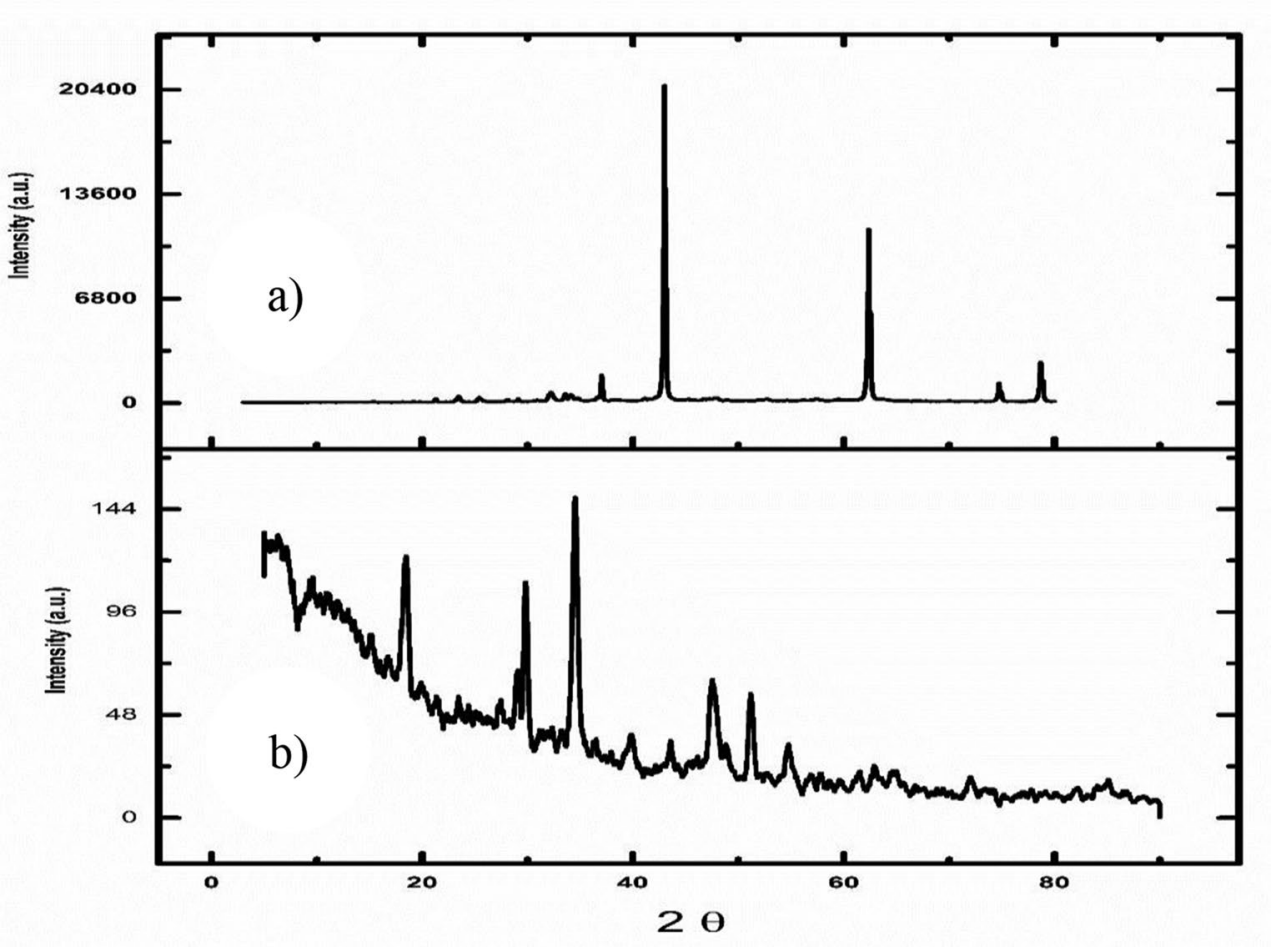
XRD patterns of the synthesized (**a**) MgO nano-catalyst and (**b**) CaO nano-catalyst

Figure 3 illustrates the X-ray diffraction (XRD) patterns of the synthesized nano-catalysts. The observed high-intensity peaks reflect a high degree of crystalline in both materials. The crystallite sizes of the CaO and MgO nanoparticles were estimated using the Debye–Scherrer equation, D = Kλ / β cosθ, yielding values of approximately 32.5 nm for CaO and 67.125 nm for MgO. The sharp and well-defined diffraction peaks further corroborate the crystalline structure and phase purity of the synthesized nano-catalysts, consistent with their catalytic functionality in the transesterification process.

#### Evaluation of CaO and MgO catalysts

The results confirm the successful synthesis of nanostructured CaO and MgO catalysts with enhanced physicochemical properties. Specifically, the BET surface areas of nano-CaO (80.69 m²/g) and nano-MgO (60.2 m²/g) reported in this study are substantially higher than those of their bulk counterparts, typically in the range of 23–28 m²/g and 19–22 m²/g, respectively [1, 2]. Moreover, the smaller crystallite size of MgO (32.5 nm) compared to that of CaO (67.125 nm) suggests superior dispersion of the MgO catalyst, which likely contributes to its enhanced catalytic activity in the transesterification process. These findings demonstrate that the precipitation–calcination synthesis route adopted in this work effectively produces highly efficient nano-catalysts, aligning with trends reported in prior studies [3, 4].

### Biodiesel characterization, yield, and conversion efficiency

**Fig. 4:**
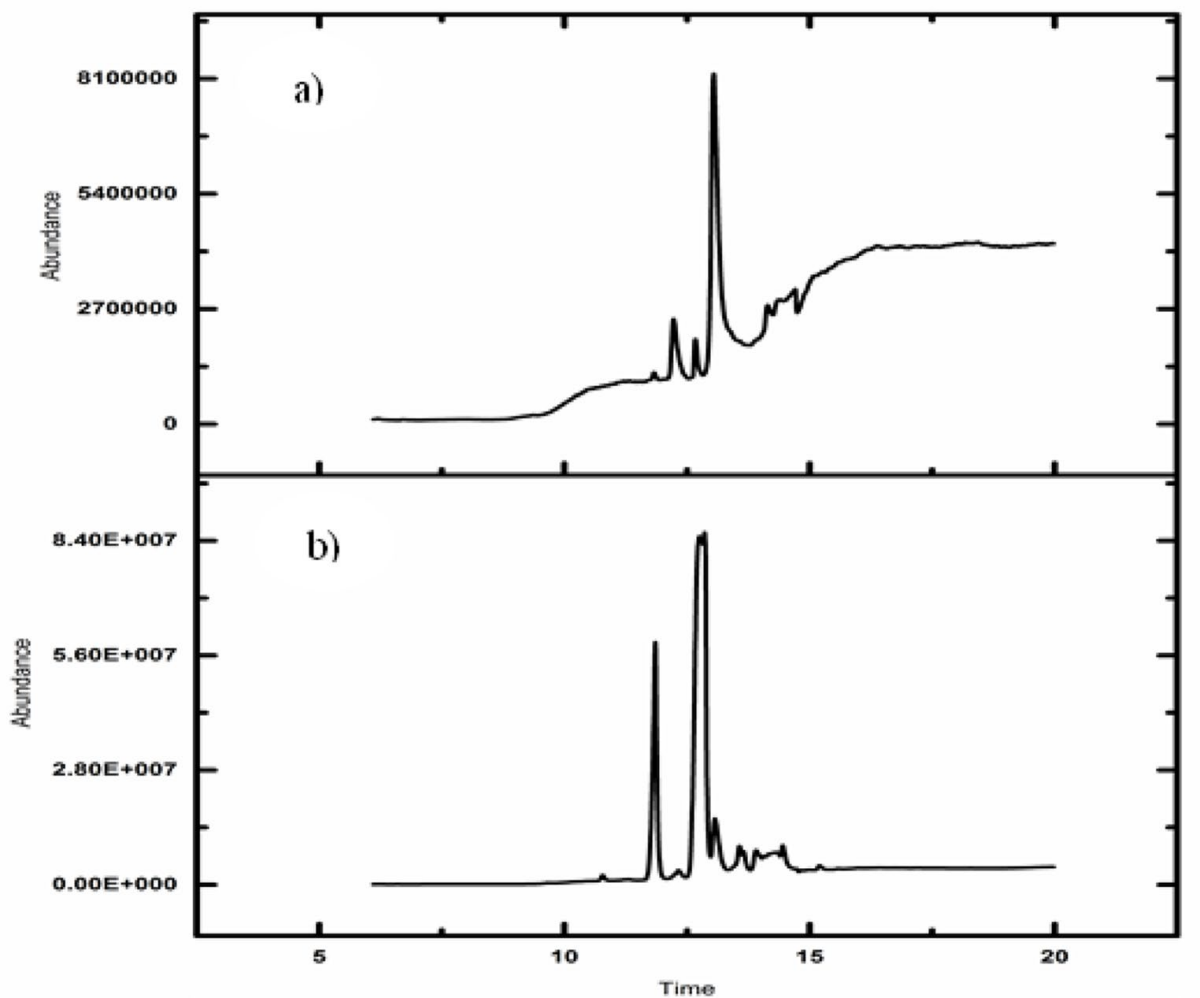
GC–MS chromatograms of biodiesel produced using (**a**) nano-MgO catalyst and (**b**) nano-CaO catalyst

Figure 4 illustrates the GC–MS fragmentation patterns of fatty acid methyl esters (FAME) in the synthesized biodiesel. For biodiesel produced using nano-MgO, the predominant methyl esters identified were 9,17-octadecadienal (Z)-methyl ester (16.3%), 9,12-octadecadienoic acid (Z, Z)-methyl ester (57.5%), and n-hexadecenoic acid methyl ester (8.8%). In contrast, biodiesel synthesized with nano-CaO was composed primarily of 9,12-octadecadienoic acid (Z, Z)-methyl ester (79%), hexadecenoic acid methyl ester (16%), and docosanoic acid methyl ester (3%).

The GC–MS spectral analysis confirms the efficient conversion of waste cooking oil into biodiesel, as evidenced by the high FAME content: approximately 96.3 wt% for nano-CaO-derived biodiesel and 95.3 wt% for nano-MgO-derived biodiesel. Correspondingly, the maximum biodiesel mass yields achieved were 97% and 92%, with calculated conversion efficiencies of 93.12% and 88%, respectively, as determined using Eq. (3).

### Diesel and biodiesel: physical and chemical characteristics

The physical and chemical properties of the fuel blends were evaluated according to standard ASTM methods: density (ASTM D4052), kinematic viscosity (ASTM D445), calorific value (ASTM D240), flash point (ASTM D93), and cloud point (ASTM D2500). The results are presented in Table 3, which also includes the ASTM D6751 specification limits for neat biodiesel (B100) for reference.

The neat diesel fuel (D100) exhibited a kinematic viscosity of 2.718 mm²/s, density of 829.8 kg/m³, calorific value of 45,871 kJ/kg, flash point of 67.5 °C, and cloud point of − 5.2 °C. Biodiesel produced using the nano-CaO catalyst (B100) demonstrated higher viscosity (5.629 mm²/s), density (898 kg/m³), and flash point (96 °C), along with a lower calorific value (39,098 kJ/kg) and increased cloud point (–0.2 °C). Similar trends were observed for the blends B10, B20, and B30, which showed incremental increases in viscosity and density with rising biodiesel content, while calorific value decreased slightly.

Biodiesel synthesized with the nano-MgO catalyst exhibited slightly higher viscosity and density compared to the CaO-based biodiesel (B100: 6.234 mm²/s, 937 kg/m³) and a lower calorific value of 38,630 kJ/kg. Correspondingly, the MgO-based blends (B10–B30) reflected these properties proportionally.

These results align with the typical behavior of biodiesel-diesel blends, where increasing biodiesel proportion leads to higher viscosity, density, and flash point, while slightly reducing energy content. Notably, all blends up to B30 remained well within ASTM D6751 limits for key parameters.

**Table 3:** Physical and chemical properties of diesel and biodiesel blends

Fuel	Kinematic Viscosity mm^2^/sec at 40 °C	Density, kg/m^3^ at 15 °C	Calorific Value kJ/kg	Flash Point, °C	Cloud Point, °C
	ASTM D-445	ASTM D-4052	ASTM D-240	ASTM D-93	ASTM D 2500
ASTM D6751 limit (B100)	1.9-6.0	800–900	37234.19	60	-12
D100	2.718	829.8	45,871	67.5	-5.2
B100 (CaO)	5.629	898	39,098	96	-0.2
B10 (CaO)	3.03	836.6	45143.9	70.6	-4.66
B20 (CaO)	3.338	843.5	44428.7	73.6	-4.14
B30 (CaO)	3.64	850.3	43724.9	76.5	-3.62
B100 (MgO)	6.234	937	38,630	90	-6.8
B10 (MgO)	3.11	840.5	45063.7	70	-5.38
B20 (MgO)	3.492	851.3	44276.8	72.5	-5.55
B30 (MgO)	3.865	861.9	43509.5	74.8	-5.72

### Impact of biodiesel blend on a diesel engine performance

The impact of biodiesel–diesel blends on engine performance was assessed using a four-stroke, single-cylinder, air-cooled, direct-injection diesel engine. The evaluation focused on key performance indicators: brake thermal efficiency (BTE), brake-specific fuel consumption (BSFC), exhaust gas temperature (Texh.), and air–fuel ratio (A/F). These parameters collectively provide a comprehensive understanding of the engine’s energy conversion efficiency, fuel utilization, combustion behavior, and thermal characteristics under different biodiesel blend ratios. The results offer valuable insights into the operational feasibility and performance implications of substituting conventional diesel with biodiesel at various proportions.

#### Brake specific fuel consumption (BSFC)

**Fig. 5:**
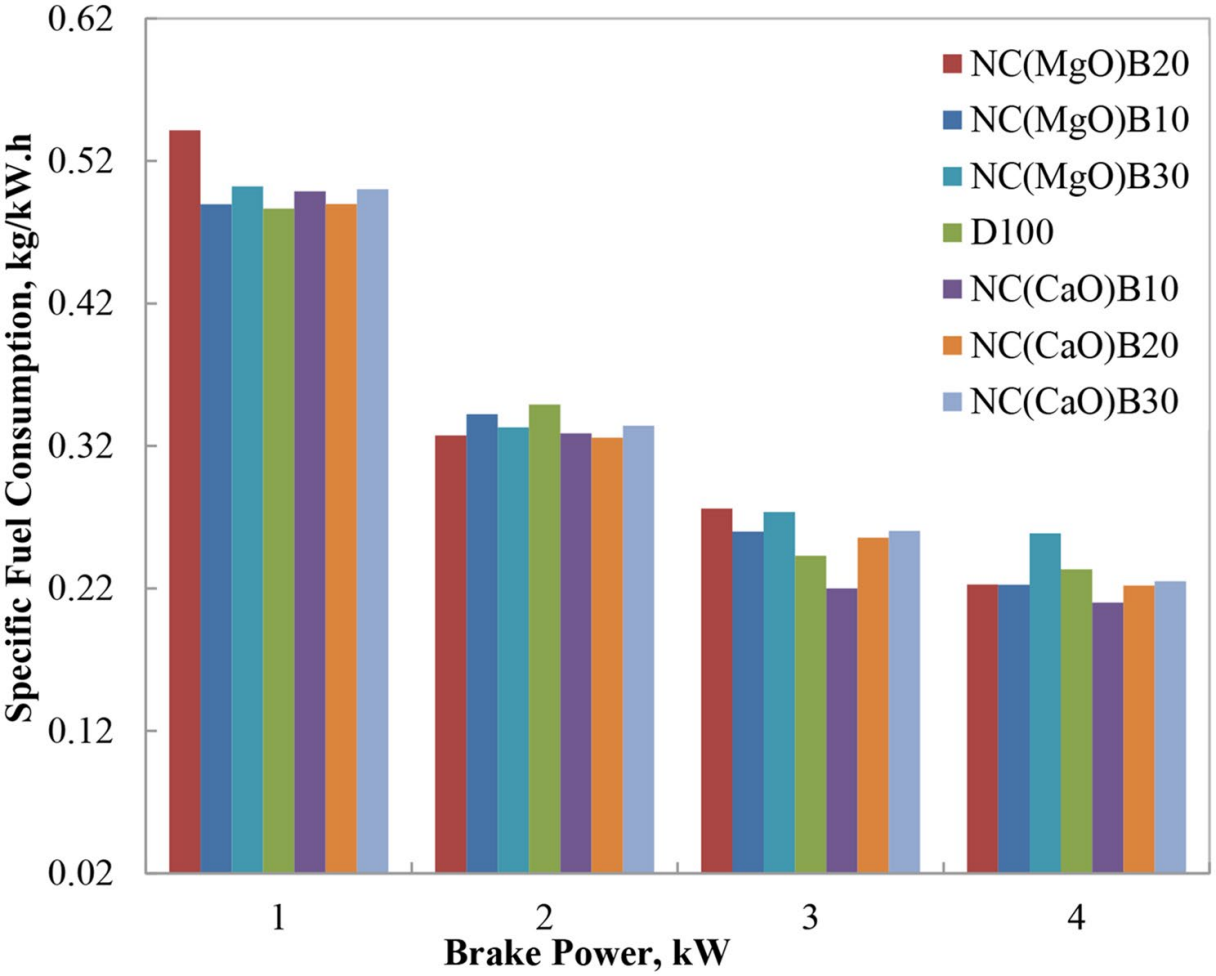
Variation of BSFC with brake power for biodiesel blends

Figure 5 illustrates the variation in brake-specific fuel consumption (BSFC) with engine load for different biodiesel blends. BSFC generally decreased as engine load increased, reflecting improved fuel utilization and higher thermal efficiency at elevated loads. However, the use of biodiesel blends, particularly those with higher viscosities and lower calorific values, resulted in higher BSFC compared to neat diesel fuel.

Specifically, biodiesel blends formulated with nano-MgO catalysts, such as NC(MgO)B20 (20% biodiesel) and NC(MgO)B30 (30% biodiesel), exhibited noticeably higher BSFC at 75% engine load. This was primarily attributed to the higher viscosity and lower calorific value of these blends relative to diesel, which impaired atomization and vaporization, leading to incomplete combustion [27]. At 75% load, NC(MgO)B20 recorded a BSFC of 0.311 kg/kWh, representing a 16.6% increase over diesel (0.267 kg/kWh). This outcome aligns with the calorific value and viscosity data, where NC(MgO)B20 measured 44,276.8 kJ/kg and 3.492 mm²/s, respectively, compared to diesel’s 45,871 kJ/kg and 2.718 mm²/s.

In contrast, the NC(CaO)B10 blend (10% biodiesel with nano-CaO catalyst) demonstrated superior performance, achieving a BSFC of 0.245 kg/kWh at 75% load—an 8.3% reduction relative to diesel. This improvement is attributed to the blend’s higher calorific value and lower viscosity, which promoted finer atomization and more complete combustion. The catalytic properties of residual CaO further enhanced oxidation reactions, contributing to improved fuel efficiency.

Overall, all tested biodiesel blends exhibited higher BSFC than diesel, except for the NC(CaO)B10 blend, which outperformed diesel. These findings corroborate prior research, which has shown that low to medium biodiesel blending ratios, particularly those incorporating nanostructured catalysts, can reduce BSFC by improving combustion efficiency and facilitating better fuel–air mixing [1–5].

#### Break thermal efficiency (BTE)

**Fig. 6:**
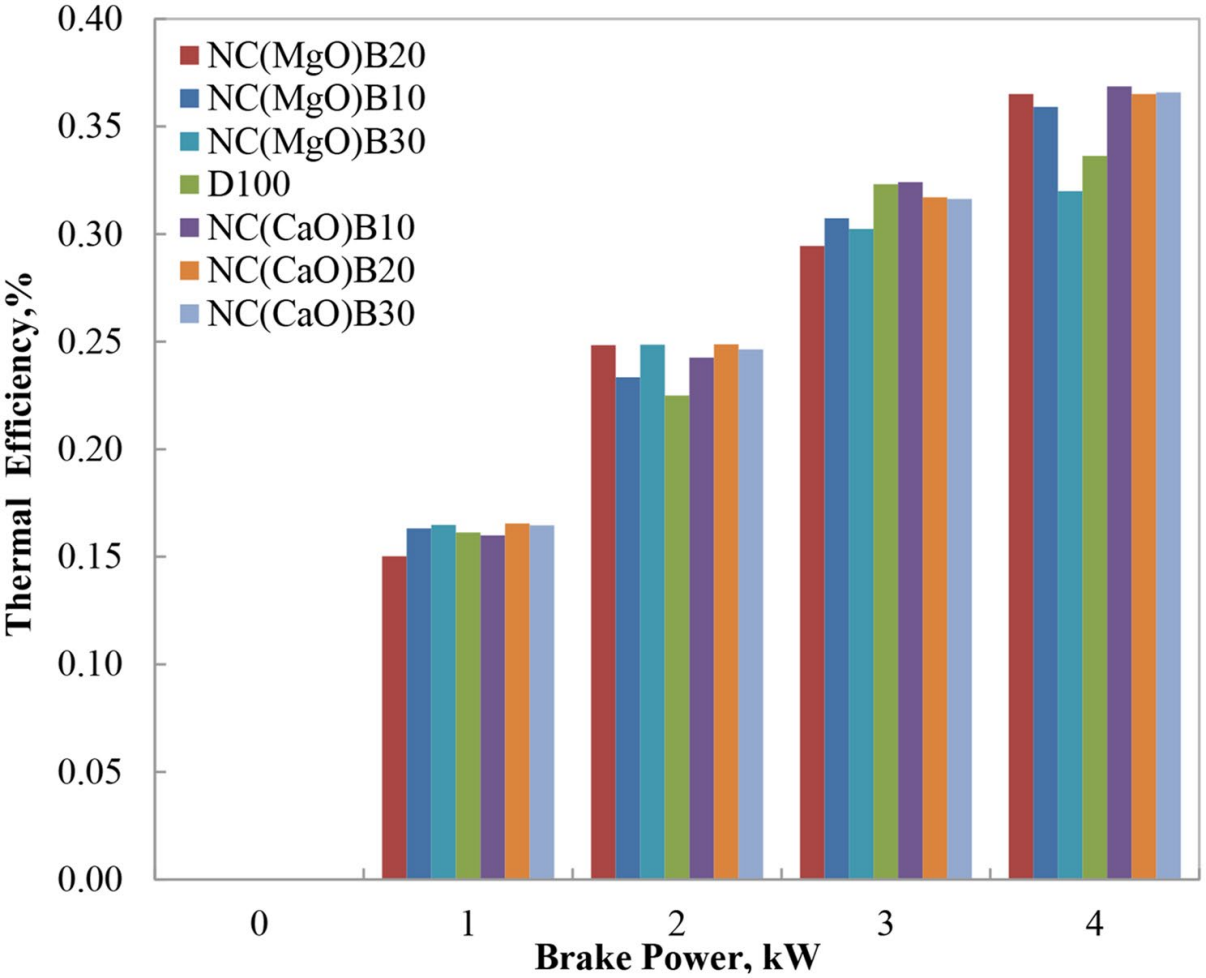
Variation of BTE with brake power for biodiesel blends

Brake thermal efficiency (BTE) quantifies the ability of the engine to convert the chemical energy of fuel into useful mechanical work. Figure 6 illustrates that BTE increases with engine load across all tested fuels, primarily due to enhanced combustion efficiency at higher loads. Biodiesel blends, however, generally exhibit slightly lower BTE than neat diesel, owing to their lower volatility, higher viscosity, greater density, and reduced calorific value. These properties tend to impair atomization and vaporization, thereby limiting the extent of combustion.

Despite these limitations, certain biodiesel blends demonstrated BTE values approaching or even exceeding those of diesel at high engine loads. This improvement is attributed to the oxygen content inherent in biodiesel, which promotes more complete combustion and reduces ignition delay, as well as the catalytic influence of the nano-structured materials. Notably, the nano-CaO blends (NC(CaO)B10, NC(CaO)B20, and NC(CaO)B30) exhibited the highest BTE values, particularly at 75% and full engine load. The elevated cylinder temperatures under these conditions enhance vaporization, facilitate better air–fuel mixing, and shorten ignition delay [28, 29].

At full engine load, the NC(CaO)B10 blend achieved a BTE of 31.2%, compared to 29.5% for diesel—representing an approximate 5.8% improvement. This enhancement is ascribed to improved fuel–air mixing, rapid volatile release, and superior thermal propagation, facilitated by the high surface reactivity of the CaO nano-catalyst (80.69 m²/g, Table (2). These effects are consistent with thermodynamic predictions for oxygenated, low-viscosity fuels, which support more favorable heat release profiles.

The oxygenated nature of biodiesel blends, combined with the catalytic activity of residual nano-CaO, further supports efficient combustion, leading to higher flame temperatures and improved thermal efficiency. The present findings align with those reported by Yesilyurt, Agarwal, and others [1–6], who observed enhanced BTE with oxygen-rich and nano-catalyzed biodiesel blends.

#### Exhaust gas temperature (T_exh_.)

**Fig. 7:**
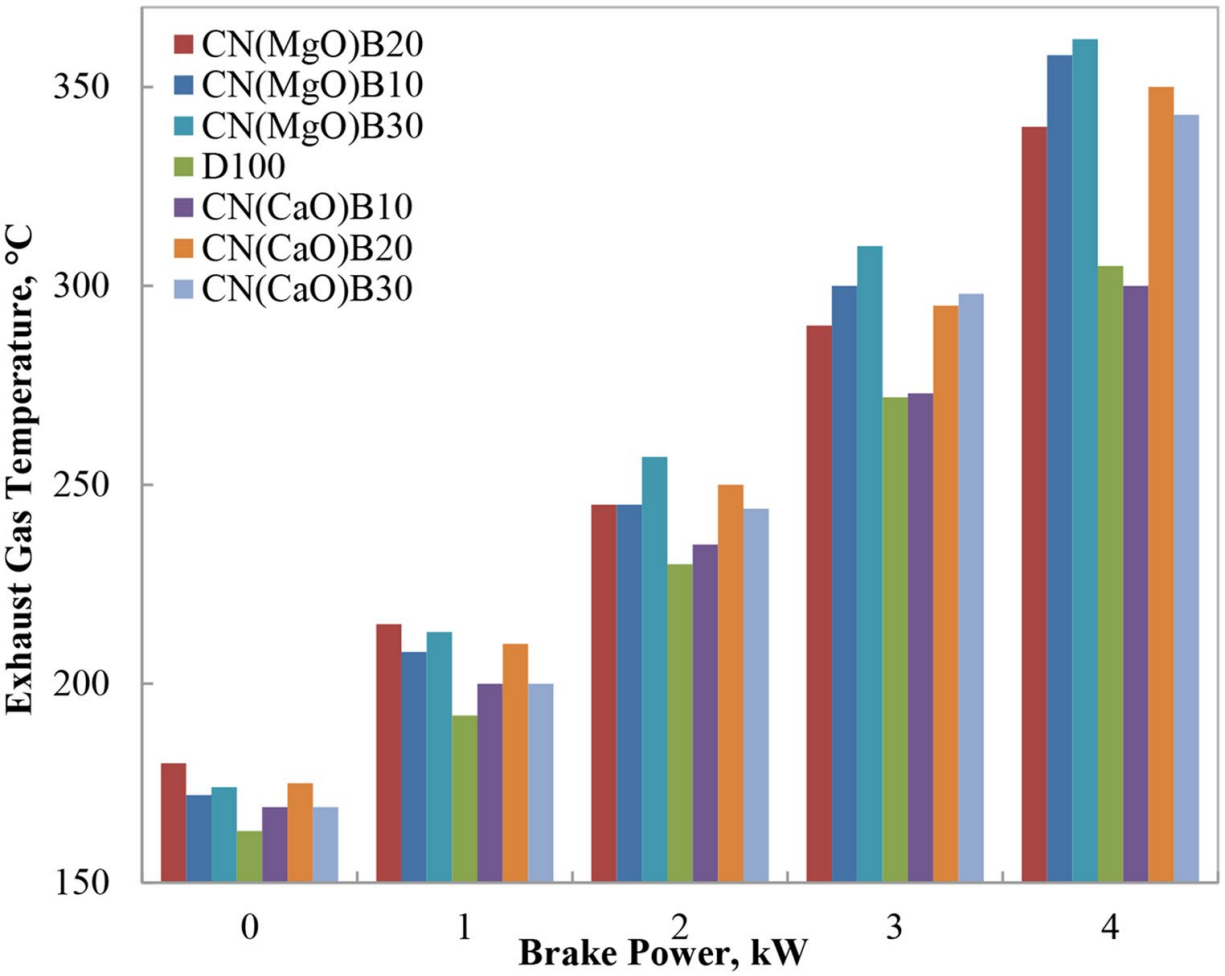
Variation of exhaust gas temperature with brake power for biodiesel blends

The variation of exhaust gas temperature (EGT) with engine load for different biodiesel–diesel blends is presented in Fig. 7. As expected, EGT increased progressively with rising engine load, attributable to the higher fuel consumption and elevated in-cylinder temperatures required to meet the increased power demand. Across all blends, the oxygen content of biodiesel contributed to more complete combustion, generally resulting in higher EGT compared to neat diesel.

At 75% engine load, the blends NC(MgO)B10, NC(MgO)B20, NC(MgO)B30, NC(CaO)B20, and NC(CaO)B30 exhibited EGT values noticeably higher than diesel, reflecting the intensified combustion facilitated by the additional oxygen in biodiesel. However, the NC(CaO)B10 blend demonstrated EGT levels comparable to diesel at intermediate loads, likely due to its lower viscosity, which enhanced fuel atomization and vaporization, thereby supporting efficient combustion without excessive heat release. Notably, at full engine load, NC(CaO)B10 showed a slightly lower EGT than both diesel and the other biodiesel blends. This suggests that improved atomization and complete combustion, supported by the catalytic effects of residual nano-CaO, mitigated excessive thermal buildup.

The modest increase in EGT with higher biodiesel content is consistent with the oxygenated nature of biodiesel, which promotes more vigorous oxidation and higher flame temperatures. Nevertheless, the observed temperatures remained within acceptable operational limits, indicating no adverse thermal stress on engine components. These findings corroborate earlier studies reporting higher EGT in engines fueled with oxygen-rich biodiesel and nano-catalyst-enhanced blends [1–5].

EGT is a critical parameter reflecting the combustion dynamics and thermal behavior within the engine cylinder. Elevated EGT in biodiesel blends, while beneficial for combustion completeness, also has implications for the durability of engine components and aftertreatment systems, such as catalytic converters. The present results align with the literature, which consistently reports higher EGT in biodiesel blends due to enhanced oxidation kinetics and improved heat release [1, 2].

#### Air-fuel ratio (A/F)

**Fig. 8:**
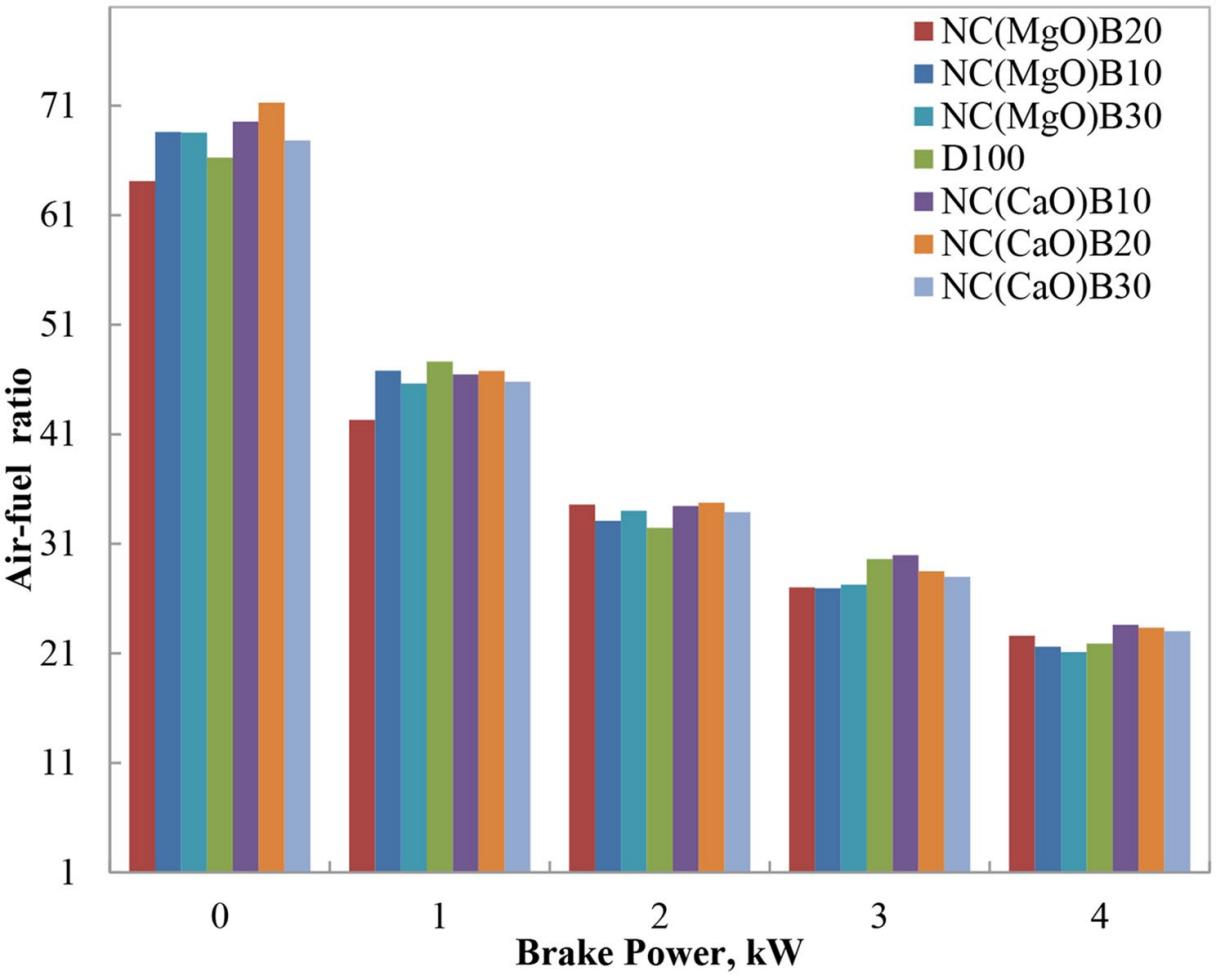
Variation of air–fuel ratio with brake power for biodiesel blends

Figure 8 illustrates the variation of air–fuel (A/F) ratio with engine load for diesel and biodiesel blends. At low engine loads, all fuels—including neat diesel, biodiesel, and biodiesel–diesel blends—exhibited relatively high A/F ratios, indicative of lean combustion conditions. As engine load increased, A/F ratios decreased across all samples, reflecting the higher fuel demand and reduced air excess at higher power outputs.

The oxygenated nature of methyl esters in biodiesel reduces the amount of external oxygen required for complete combustion, thereby lowering the A/F ratio relative to neat diesel. Most biodiesel blends demonstrated lower A/F ratios than diesel, attributable to their higher fuel consumption and intrinsic oxygen content. Notably, the NC(CaO)B10 blend maintained an A/F ratio nearly identical to that of diesel at 75% engine load, highlighting its favorable chemical and physical properties that promote efficient combustion without excessive fuel enrichment. Conversely, the NC(MgO)B10 blend exhibited the largest deviation from diesel, with a 7.1% lower A/F ratio at 75% load, likely due to its higher viscosity and reduced calorific value compared to diesel.

Interestingly, the CaO-catalyzed B10 and B20 blends demonstrated slightly higher A/F ratios than diesel at lower and medium loads, suggesting leaner combustion resulting from improved atomization and the oxygenated structure of biodiesel. This trend is consistent with earlier reports on biodiesel–diesel blends synthesized through both conventional and nano-catalyzed routes [1–4]. The leaner combustion of these blends is advantageous in reducing smoke emissions while maintaining stable engine operation.

### Impact of biodiesel blends on diesel engine exhaust emissions

The effects of biodiesel–diesel blends on exhaust emissions were evaluated using a four-stroke, single-cylinder, air-cooled, direct-injection diesel engine. Key emission parameters analyzed included carbon monoxide (CO), carbon dioxide (CO₂), and oxygen (O₂) concentrations in the exhaust gas. These metrics provide critical insights into the combustion completeness, environmental performance, and excess air utilization associated with various biodiesel blend ratios.

#### CO_2_ emissions

**Fig. 9:**
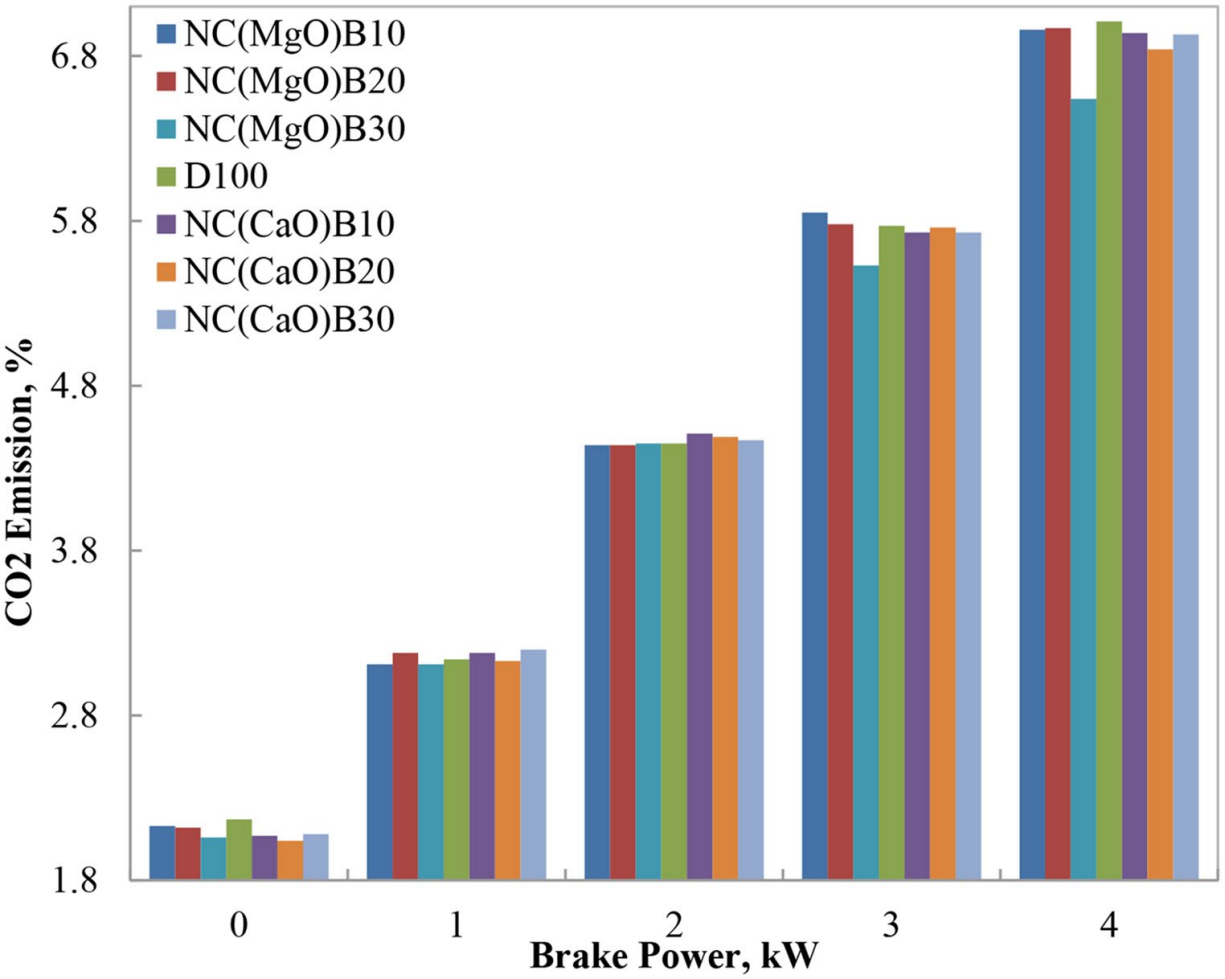
CO₂ concentrations at different brake power levels for biodiesel blends

Carbon dioxide (CO₂) emissions, a major contributor to global warming, serve as a reliable indicator of combustion completeness within the engine cylinder. Figure 9 illustrates the variation of CO₂ emissions for biodiesel–diesel blends as a function of brake output power. At low engine loads, CO₂ emissions from both diesel and biodiesel blends remained relatively low due to incomplete combustion and reduced volumetric fuel consumption. As engine load increased, CO₂ emissions rose correspondingly, reflecting improved combustion and higher fuel throughput.

At 75% engine load, the biodiesel blend incorporating NC(MgO)B30 exhibited a CO₂ reduction of 4.2% compared to neat diesel, while NC(MgO)B20 and NC(MgO)B10 showed marginal increases of 0.5% and 1.6%, respectively. These modest increases at lower blend ratios are likely attributable to fuel heterogeneity and suboptimal atomization at intermediate oxygen levels, which can enhance air utilization and raise combustion temperatures. Conversely, the NC(MgO)B30 blend’s superior performance likely stems from its improved oxygen content and catalytic properties, which facilitate more complete combustion at higher loads and temperatures.

Our findings are consistent with Ashok et al. [12], who reported a 3–5% decrease in CO₂ emissions for MgO-catalyzed waste cooking oil (WCO) biodiesel at comparable blend ratios. The slightly greater reduction observed here may be attributed to the use of nano-MgO with higher surface area and better-controlled oxygen delivery. Variability in reported results across literature often arises from differences in catalyst crystallinity, feedstock composition, and engine configurations. For example, Banković–Ilić et al. [10] demonstrated that nano-CaO particles smaller than 70 nm promoted more complete combustion, aligning with our observation of high oxygen content in NC(CaO)B30 (~ 18.5%) and concurrent reductions in CO emissions.

In summary, biodiesel blends produced lower CO₂ emissions than neat diesel, with NC(MgO)B30 achieving the most significant reduction of 4.2%. This reduction can be attributed to the combined effects of enhanced combustion efficiency, the lower carbon-to-hydrogen ratio of biodiesel, and the catalytic activity of nanostructured oxides. Similar trends have been reported in previous studies, particularly when nanocatalysts improve fuel atomization and oxidation kinetics [1–3].

#### CO emissions

**Fig. 10:**
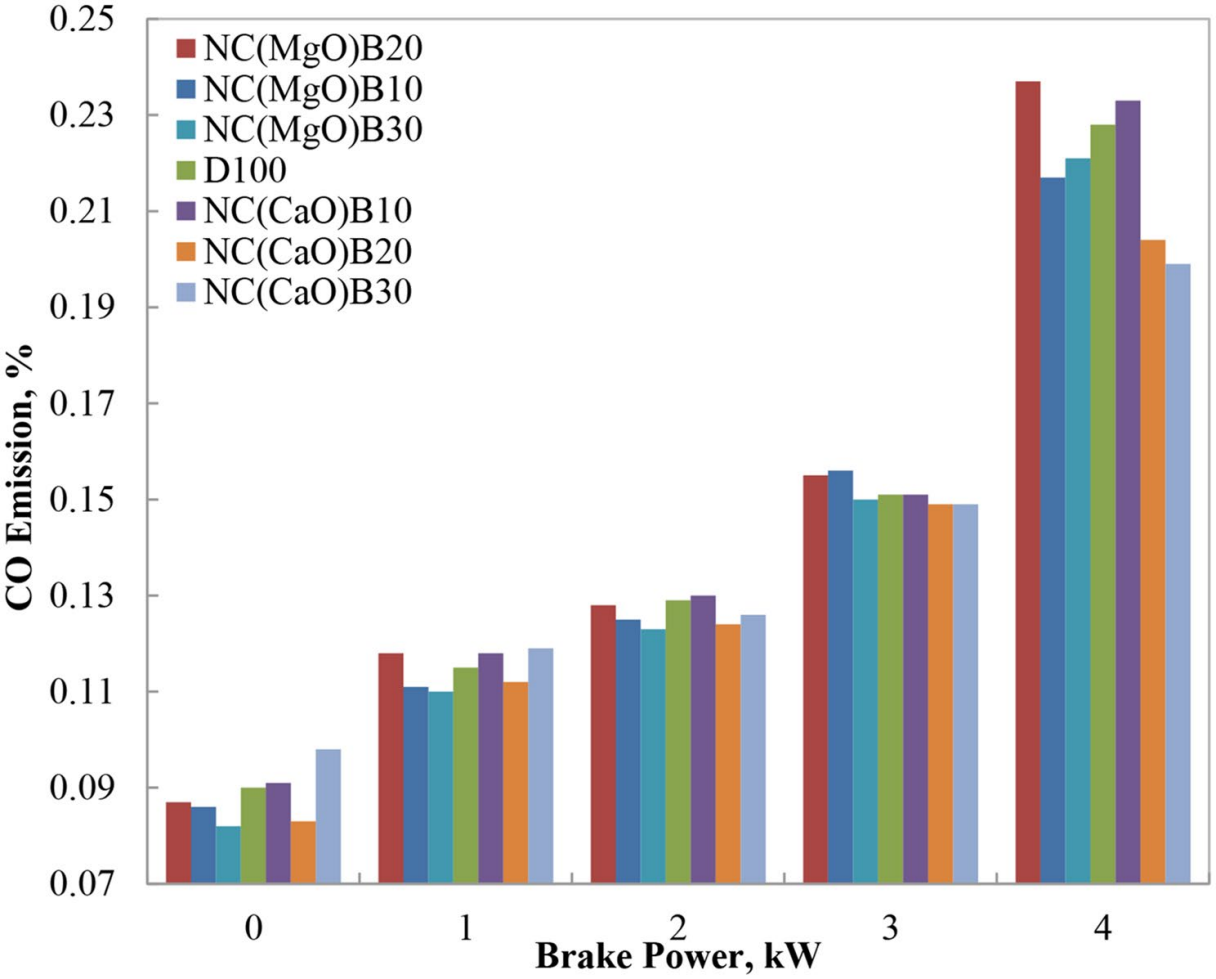
CO emissions at different brake power levels for biodiesel blends

Figure 10 illustrates the variation of carbon monoxide (CO) emissions with engine brake power for the tested biodiesel–diesel blends. CO emissions primarily result from incomplete combustion, typically due to insufficient oxygen availability within the combustion chamber [30]. In biodiesel blends, however, the inherent oxygen content of the methyl ester promotes more complete combustion, thereby reducing CO emissions relative to neat diesel.

At 75% engine load, the biodiesel blends incorporating NC(CaO)B20 and NC(CaO)B30 demonstrated the most substantial reduction in CO emissions, achieving approximately a 0.7% decrease compared to pure diesel. This improvement is attributed to the enhanced oxidation reactions facilitated by both the oxygenated biodiesel molecules and the catalytic effect of the nano-CaO additive. In contrast, biodiesel blends containing NC(MgO)B10 and NC(MgO)B20 exhibited CO emissions that were approximately 3.3% higher than diesel at the same load. These elevated emissions are indicative of richer mixtures, likely stemming from suboptimal atomization and combustion kinetics at these blend ratios.

Overall, the use of biodiesel blends—particularly those catalyzed with nano-CaO—led to markedly lower CO emissions compared to neat diesel. This outcome highlights the dual role of biodiesel’s oxygen content and the catalytic activity of nanostructured oxides in promoting more complete oxidation of the fuel. These findings are consistent with previous reports that document CO reductions in biodiesel–diesel blends, especially when enhanced by Nano catalys.

#### Oxygen concentration

**Fig. 11:**
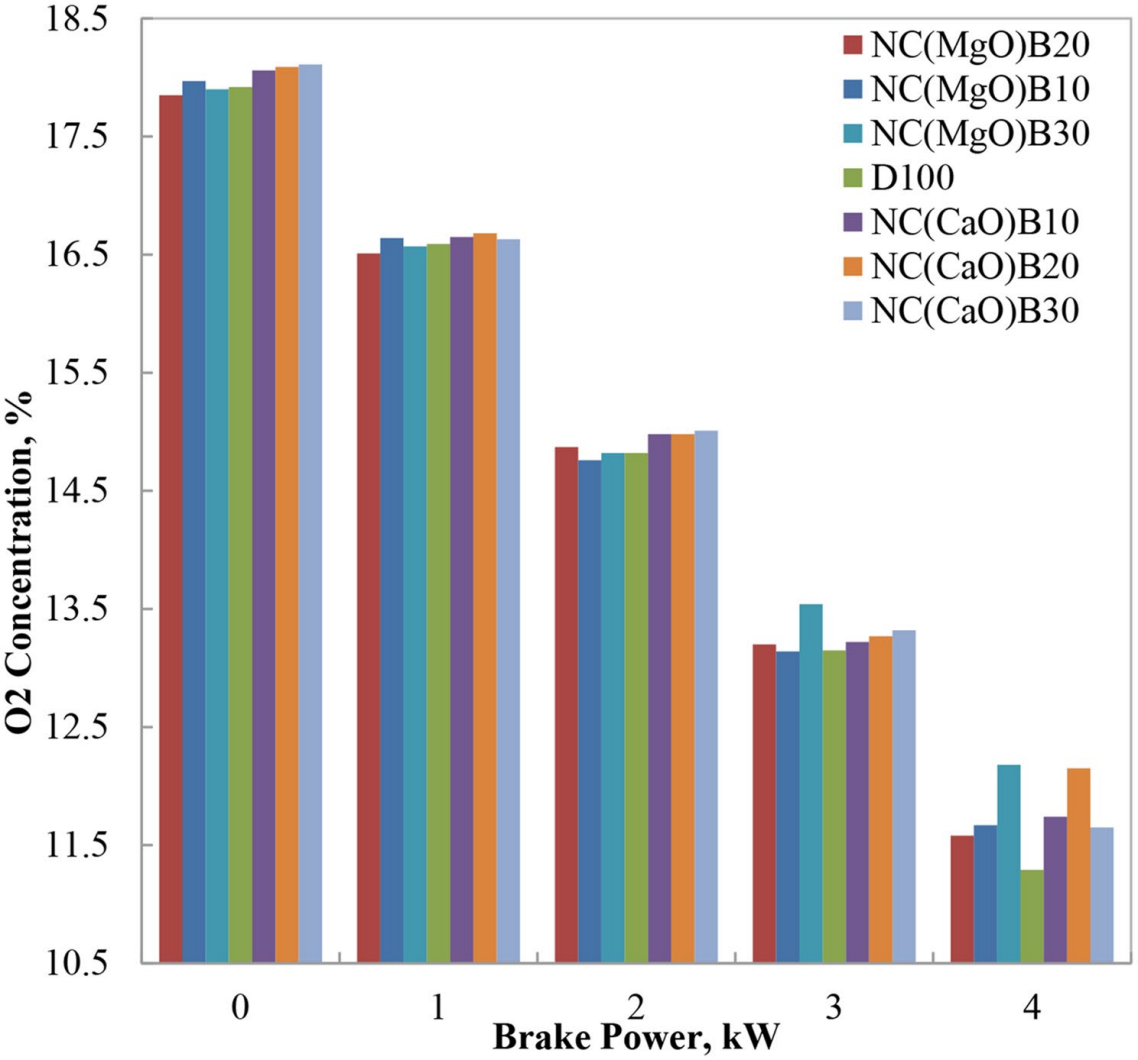
Variation of O₂ concentration at different brake power levels for biodiesel blends

Figure 11 illustrates the variation in exhaust oxygen concentration with engine brake power for both neat diesel and biodiesel–diesel blends. At low engine loads, higher oxygen concentrations were observed for all fuels, attributable to the richer mixture and excess air present in the combustion chamber under these conditions. As engine load increased, oxygen levels in the exhaust decreased due to higher fuel demand and more complete consumption of available oxygen.

The elevated oxygen content of methyl ester fuels and their blends—stemming from the oxygen atoms incorporated within their molecular structure—accounts for the consistently higher exhaust oxygen concentrations observed for biodiesel blends compared to neat diesel. Among the tested fuels, the blend containing NC(CaO)B30 exhibited the highest exhaust oxygen concentration at 75% load, approximately 3% higher than diesel. This elevated oxygen availability facilitates more complete oxidation of the fuel, which is reflected in the correspondingly lower CO emissions reported for the same blend.

These findings confirm that biodiesel blends enhance the oxygen content in the combustion chamber and exhaust gases, contributing to improved combustion efficiency and leaner exhaust mixtures. Notably, the CaO-catalyzed B20 and B30 blends demonstrated superior oxygen enrichment, consistent with previous studies reporting similar trends for oxygenated fuels and nanocatalyst-enhanced biodiesel blends.

### Performance and emissions comparative analysis

This section presents a consolidated comparison of engine performance and emissions between nano-catalyzed biodiesel blends and neat diesel. Among the tested fuels, the B10 blend catalyzed by nano-CaO exhibited the most favorable performance characteristics, achieving higher brake thermal efficiency and lower specific fuel consumption relative to both neat diesel and higher biodiesel blends. This suggests that NC(CaO)B10 strikes an optimal balance between energy content and combustion quality.

Although B30 blends demonstrated notable reductions in CO₂ emissions—most prominently with NC(MgO)B30 achieving a 4.2% reduction compared to diesel—these blends also exhibited increased fuel consumption and slightly reduced brake power. These findings are consistent with previous studies [1, 2], which reported that higher biodiesel content tends to improve emissions at the expense of efficiency.

From a practical perspective, NC(CaO)B10 appears to offer the best compromise between engine performance, emissions mitigation, and operational compatibility with conventional diesel engines. MgO-catalyzed blends, while generally less efficient than their CaO counterparts, may still provide advantages in applications where stringent emissions regulations are a priority.

To further validate these findings, future studies should investigate long-term engine durability and conduct comprehensive economic and lifecycle assessments to support potential large-scale deployment.

At 75% engine load, the comparative evaluation of NC(CaO)B10, B20, B30 and NC(MgO)B10, B20, B30 against D100, NC(CaO)B100, and NC(MgO)B100 confirmed the superior performance of NC(CaO)B10. Its lower viscosity and higher calorific value contributed to improved atomization and vaporization, thereby enhancing combustion efficiency and reducing emissions.

## Conclusions

This study investigated the production of biodiesel from waste cooking oil via nano-catalyzed transesterification. Both nano-calcium oxide (NC–CaO) and nano-magnesium oxide (NC–MgO) catalysts demonstrated high purity, nanoscale crystallinity, large specific surface areas, and distinct particle morphologies, confirming their effectiveness for biodiesel synthesis. XRD and GC analyses revealed that NC–CaO achieved superior biodiesel conversion efficiency compared to NC–MgO. The resulting biodiesel complied with ASTM D6571 specifications, and blending with diesel improved critical fuel properties, including density, viscosity, calorific value, and cloud point.

In engine performance and emissions evaluations, the NC–CaO B10 blend exhibited the most advantageous balance, reducing brake-specific fuel consumption (BSFC) by 8.3%, enhancing brake thermal efficiency (BTE), and maintaining exhaust gas temperature and air–fuel ratio comparable to conventional diesel. The NC–MgO B30 blend achieved the largest CO₂ emissions reduction (4.2%), while NC–CaO B20 and NC–CaO B30 significantly lowered CO emissions. Furthermore, NC–CaO B30 demonstrated the highest oxygen content, supporting more complete combustion.

These findings highlight the promise of nano-CaO-catalyzed biodiesel blends, particularly B10, as a viable and sustainable alternative for diesel engines, offering improved performance and reduced emissions. Future research should investigate higher blend ratios (e.g., B40–B50) and explore dual nano-catalyst systems to further optimize fuel properties and engine performance.

## Nomenclature and symbols

**Table:** 

Symbol	Description	Unit
B	Biodiesel	-
BXX	Biodiesel blend with XX% biodiesel	%
CI	Compression Ignition	-
CO	Carbon Monoxide	%
CO₂	Carbon Dioxide	%
CaO	Calcium Oxide	-
MgO	Magnesium Oxide	-
EGT	Exhaust Gas Temperature	°C
GC-MS	Gas Chromatography-Mass Spectrometry	-
SEM	Scanning Electron Microscopy	-
BET	Brunauer-Emmett-Teller Surface Area Analysis	-
WCO	Waste Cooking Oil	-
O₂	Oxygen	%
HC	Hydrocarbons	ppm
PM	Particulate Matter	mg/m³
η	Efficiency	%
P	Power	kW
BSFC	Brake Specific Fuel Consumption	g/kWh
BTE	Brake Thermal Efficiency	%
BSEC	Brake Specific Energy Consumption	MJ/kWh
RPM	Revolutions Per Minute	rpm
ρ	Density	kg/m³
ν	Kinematic Viscosity	mm²/s
µm	Micrometer	µm

## Data Availability

The datasets supporting the conclusions of this article are included within the article.
